# Cochlear implant surgery using the 4 K-three-dimensional exoscope ORBEYE: a comparative study with conventional operative microscope

**DOI:** 10.1007/s00405-025-09794-y

**Published:** 2025-11-12

**Authors:** Asato Ogata, Yoshiyuki Kawashima, Taro Fujikawa, Keiji Honda, Taku Ito, Ayako Nishio, Ayako Maruyama, Yoshimaru Mizoguchi, Takeshi Tsutsumi

**Affiliations:** Department of Otolaryngology, Institute of Science Tokyo, 1-5-45 Yushima, Bunkyo-Ku, Tokyo, 113-8519 Japan

**Keywords:** Exoscope, Cochlear implant, Operating microscope, Otologic surgery

## Abstract

**Purpose:**

To evaluate the feasibility of the 4 K-three-dimensional (3D) exoscope ORBEYE (ExS) in cochlear implant (CI) surgery and compare its advantages and limitations with the conventional operating microscope (OM).

**Methods:**

We retrospectively analyzed 77 consecutive CI surgeries performed between January 2019 and December 2023 (42 with ExS and 35 with OM). Surgical outcomes, complications, and hearing results were compared between the groups. Eight otologists completed a questionnaire comparing technical performance, operative performance, team interaction, and procedure-specific performance of both visualization systems.

**Results:**

All ExS group surgeries were successfully completed using the ExS alone. The operating time was significantly longer in the ExS group than in the OM group (162 vs. 130 min, *p* = 0.0051), and the resident participation rate in mastoidectomy procedures was higher (28.6% vs. 8.6%, *p* = 0.0417). When excluding cases where residents performed mastoidectomy, the operating time did not markedly differ between the groups (152 vs. 130.5 min, *p* = 0.1532). The hospital stay and complication rates were also comparable. Both systems achieved similar hearing improvements. The questionnaire results revealed a clear preference for ExS across all evaluation domains except for natural stereoscopic perception. ExS demonstrated advantages in implant bed creation, cochleostomy, and electrode insertion.

**Conclusions:**

The 4 K-3D ExS ORBEYE demonstrated comparable surgical outcomes to those of conventional OM in CI surgery. The longer operative time in the ExS group appeared to reflect educational benefits rather than technical limitations. ExS offers a viable alternative to OM for CI surgery, with advantages across multiple performance domains, including team interaction and surgical education.

## Introduction

In 1961, House et al. performed the first cochlear implant (CI) surgery via the facial recess approach and round window insertion using a binocular operating microscope (OM) [1]. Since then, the surgical procedure for CI has remained largely unchanged, and OM has continued to serve as the gold-standard visualization tool for CI surgery. Since its introduction to the field of otologic surgery in the 1950 s, the optical performance of OM has continuously improved, providing otologic surgeons with a bright field of view at high resolution, even at high magnification. However, OM has several shortcomings, such as limited visibility in the surgical fields at the bottom of narrow passages. Stereoscopic visualization of the surgical field is limited to the operator and assistant, while other operating room personnel can only observe the surgical field through two-dimensional (2D) monitors, lacking the three-dimensional (3D) perspective essential for a comprehensive understanding of surgical anatomy. Furthermore, a short working distance and limited range of motion sometimes force the surgeon and assistant to maintain poor ergonomic posture [2].

In the 2010 s, the high-definition video microscope, also known as the exoscope (ExS), emerged in the field of neurosurgery and has gradually replaced the OM as an alternative method for magnification and illumination of the surgical field [3, 4]. The video camera of the ExS is positioned above the surgical field, and images are visualized on a monitor in front of the operator. State-of-the-art ExSs provide surgeons with 4 K-3D images without recognizable latency. However, while the ExS has been increasingly used in various surgical fields, a limited number of studies have reported its application in otologic surgery [5–21], and relatively few studies have evaluated its use in CI surgery [6, 9, 13, 17, 18, 20, 21].

Given the above, we evaluated the feasibility of the ExS in CI surgery and compared its advantages and limitations with those of a conventional OM.

## Materials and methods

This study was conducted in accordance with the tenets of the Declaration of Helsinki and approved by the Institutional Review Board of Tokyo Medical and Dental University Faculty of Medicine (M2023-305). We retrospectively analyzed CI surgeries performed at our institution and compared the outcomes between the two different visualization systems.

### Visualization systems used for CI surgery

Prior to January 2019, we exclusively used a Zeiss OPMI Pentero OM (Carl Zeiss Meditec AG, Jena, Germany) for CI surgery. In January 2019, we gradually introduced the 4 K-3D ExS system (ORBEYE™; Sony Olympus Medical Solutions Inc., Tokyo, Japan), initially for select cases and subsequently for all CI surgeries.

### Patients

All consecutive patients who underwent CI surgery at Tokyo Medical and Dental University hospital between January 2019 and December 2023, with a minimum follow-up period of 12 months after surgery, were included in this study. Based on the visualization system used, patients were categorized into either the OM or the ExS group. Medical records were retrospectively reviewed to collect data, including the demographics (age, sex), length of hospital stay, and pre- and post-operative audiological outcomes. Operative records were retrospectively reviewed to collect data, including the visualization system used, details of the surgical procedure, surgeons, operating time, and intra- or postoperative complications. The operating time was defined as the time taken from the start of the skin incision to the end of the incision closure. Cases involving concurrent procedures (tympanoplasty) or demonstration of a new surgical assistance device utilizing virtual reality (VR) technology were excluded from the operating time analysis because the duration of these additional steps could not be determined from the operative records.

### The 4 K-3D ExS system and the operating room layout

**Fig. 1:**
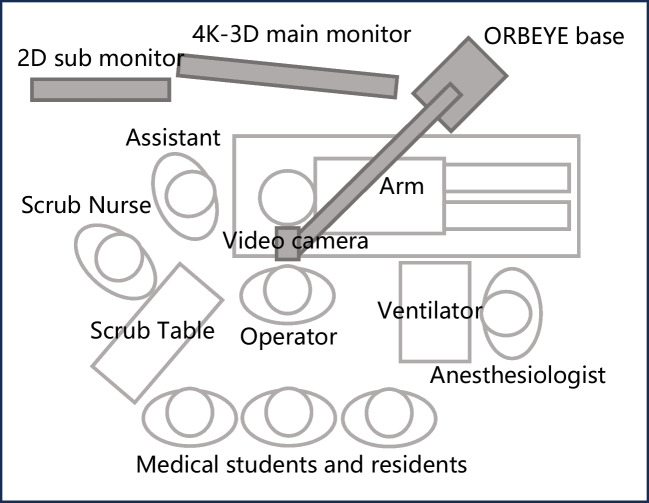
Operating room layout for the 4 K-3D exoscope system showing the positioning of the ORBEYE base, arm, and video camera relative to the surgical team members (operator, assistant, scrub nurse, anesthesiologist) and equipment (4 K-3D main monitor, 2D sub monitor, scrub table, ventilator)

The ExS system consists of a main unit, a 55-inch 4 K-3D monitor, and a 32-inch sub-monitor. The main unit features a 4 K-3D digital video camera mounted on a flexible arm extending from the base, which is equipped with an LED light source and an image processor. Figure 1 illustrates the arrangement of the surgical equipment and team members in the operating room. The base of the ExS system is diagonally positioned at the foot end of the operating table across the surgeon. The flexible arm extends across the operating table towards the operator, positioning the camera above the surgical field. Unlike the standard configuration, where the camera is suspended below the arm, we mounted the camera above the arm, which prevented the arm from obstructing the monitor and enhanced monitor visibility for the operator. The 4 K-3D main monitor is positioned approximately 1.5 m directly opposite the operator and slightly rotated toward the head end of the operating table. This position enables stereoscopic visualization of both the operator and assistant. A 2D sub-monitor was placed adjacent to the main monitor for reference by the scrub nurse. Medical students and residents can observe the procedure by positioning themselves behind the operator while wearing 3D glasses, allowing them to share the same high-resolution 3D view displayed on the main monitor.

### Surgical procedure

All CI surgeries were performed using a standardized technique. The procedure began with a 4- to 5-cm post-auricular incision. Standard mastoidectomy was then performed using either the OM or ExS, followed by posterior tympanotomy and exposure of the round window, including removal of the round window overhang and inferior bony projection. Next, a posterior temporalis subperiosteal pocket was created and a receiver stimulator well was drilled. This procedure was performed under a standard surgical shadowless lamp in the OM group, whereas in the ExS group, the same procedure was performed while continuing to use the ExS. The receiver stimulator was inserted into the well and secured with nonabsorbable sutures. The round window membrane was opened using a fine pick, and an electrode array was inserted into the cochlea. Both groups used their respective visualization systems (OM or ExS) for this procedure. Most procedures were performed by board-certified otologic surgeon instructors of the Japanese Otological Society, but in some cases, the mastoidectomy portion was performed by residents or mid-career otologists under the direct supervision of the instructors.

### Questionnaire

**Fig. 2:**
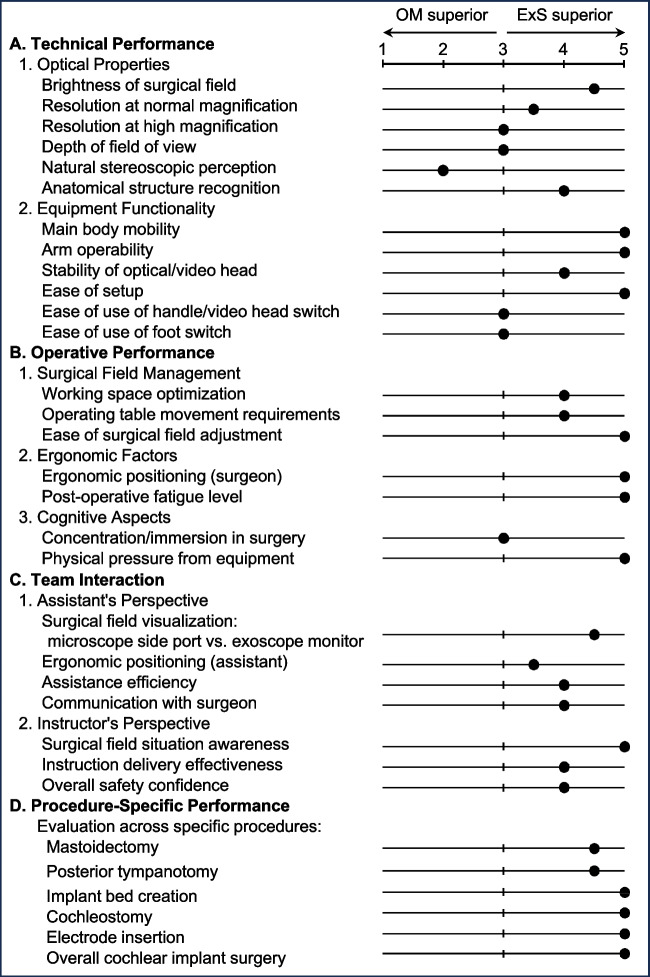
A comparison of the operating microscope (OM) and 4 K-three-dimensional exoscope (ExS) based on questionnaire responses from eight otologists evaluating technical performance, operative performance, team interaction and procedure-specific performance in cochlear implant surgery on a 5-point scale, where higher scores indicate ExS superiority

To assess the usability of the OM and ExS in CI surgery, we developed a questionnaire comprising 32 items organized into four main categories: technical performance (optical properties and equipment functionality), operative performance (surgical field management, ergonomic factors, and cognitive aspects), team interaction (assistants and instructors’ perspectives), and procedure-specific performance (Fig. 2). To evaluate the procedure-specific performance, the CI surgical procedure was divided into five stages: mastoidectomy, posterior tympanotomy, implant bed creation, cochleostomy, and electrode insertion. Each item was scored on a five-point Likert scale: 1 ("OM superior"), 2 ("OM somewhat superior"), 3 ("OM and ExS equivalent"), 4 ("ExS somewhat superior"), and 5 ("ExS superior"). Eight experienced otologists who had performed CI surgeries using both the OM and ExS completed the questionnaire based on their overall experience with both systems.

### Statistical analyses

All statistical analyses were performed using the Prism software program (ver. 10.4.1; macOS; GraphPad Software Inc., San Diego, CA, USA). Continuous variables were compared using the two-tailed Mann–Whitney U test, whereas categorical variables were compared using Fisher's exact test. For the questionnaire data analysis, the median scores were calculated for each item and plotted to visualize the comparative assessment between the OM and ExS systems. A value of p < 0.05 was considered to indicate significance.

## Results

### Patient demographics

The patient demographics, surgical outcomes, and hearing results of the ExS and OM groups are summarized in Table 1. Between January 2019 and December 2023, 77 patients underwent CI surgery at Tokyo Medical and Dental University Hospital, and all patients were followed for more than 12 months. Of these 77 patients, 42 underwent surgery using the ExS, and 35 underwent surgery using the OM.

**Table 1:** A comparison of the demographics and surgical outcomes between exoscope and operating microscope groups

	Exoscope	Operating microscope	
	n = 42	n = 35	*p* Value
Age at surgery	64.5 (54.0–75.0)	68.0 (51.0–75.0)	0.6971
Female sex (%)	29 (69.0)	20 (57.1)	0.3440
Preoperative hearing level			
PTA (dB)	97.5 (90.0–115.0)	103.3 (86.7–115.0)	0.6854
max SDS (%)	15 (5–35)	0 (0–30)	0.0881
max SDS with HA (%)	30 (20–45)	5 (0–46)	0.1618
Surgical outcomes	n = 33	n = 35	
Operating time (min)	162 (145–187)	130 (110–168.0)	**0.0051**
Mastoidectomy by residents (%)	12 (28.6)	3 (8.6)	**0.0417**
Operating time (non-resident) (min)	152 (133–162)	130.5 (111.5–165.8)	0.1532
Length of hospital stay (d)	5 (4–6)	4 (4–5)	0.0779
Complication (%)	3 (7.1)	1 (2.9)	0.3417
Postoperative hearing level			
PTA with CI (dB)	36.7 (31.3–38.8)	33.3 (30.0–36.7)	0.1700
max SDS with CI (%)	55 (28–69)	52 (30–70)	0.9453

Data are presented as the median (interquartile range) or n (%)

Statistical analyses were performed using the two-tailed Mann–Whitney U test for continuous variables and Fisher's exact test for categorical variables. A *p* value of < 0.05 was considered statistically significant

PTA, pure-tone average; max SDS, maximum speech discrimination score; HA, hearing aid; CI, cochlear implant

No significant differences were observed between the two groups in terms of the age at surgery (p = 0.6971) and sex ratio (p = 0.3440). In terms of preoperative hearing level, the pure-tone average (PTA) showed no significant difference between the groups (p = 0.6854). The maximum speech discrimination score (max SDS) with and without hearing aids also showed no significant differences between the groups (p = 0.0881 and 0.1618, respectively).

### Surgical outcomes

In both groups, CI surgery was completed using the ExS or OM alone. For the operating time analysis, nine ExS cases were excluded because of concurrent tympanoplasty (one case) and demonstration of VR assistance (eight cases). The median (interquartile range [IQR]) operating time in the ExS group (162 [145–187] min) was significantly longer than that in the OM group (130 [110–168] min, *p* = 0.0051, Table 1). In the ExS group, mastoidectomy was performed by a resident in 12 (28.6%) of the 42 operations, a significantly higher rate than in the OM group (3 [8.6%] of the 35 operations, *p* = 0.0417). When excluding the cases of mastoidectomy by residents, the operating time showed no statistically significant difference between the groups (152 [133–162] min in the ExS group and 130.5 [111.5–165.8] min in the OM group, *p* = 0.1532). The median length of hospital stay was similar between the groups (5 [4–6] days in the ExS group and 4 [4, 5] days in the OM group, *p* = 0.0779). Although the complication rate in the ExS group (7.1%) was higher than that in the OM group (2.9%), the difference was not statistically significant (*p* = 0.3417), and all complications were minor dizziness.

Both groups showed significant improvements in hearing outcomes after CI surgery. The median PTA in the ExS group improved significantly from 97.5 (90.0–115.0) dB preoperatively to 36.7 (31.3–38.8) dB with the CI postoperatively, while in the OM group, it improved from 103.3 (86.7–115.0) dB to 33.3 (30.0–36.7) dB. The degree of PTA improvement was not significantly different between the groups (*p* = 0.1700). Similarly, the median maximum SDS in the ExS group improved significantly from 15% (5%−35%) preoperatively to 55% (28%−69%) with the CI postoperatively, and in the OM group, it improved from 0% (0%−30%) to 52% (30%−70%). The degree of maximum SDS improvement also showed no significant difference between the groups (*p* = 0.9453).

### Questionnaire

Figure 2 presents a comparison between the OM and ExS based on the questionnaire responses from eight otolaryngologists. The black dots indicate median scores on the 5-point Likert scale described in the Methods section, with lower scores favoring the OM and higher scores favoring the ExS. For optical properties (Topic A1), the ExS was rated superior in brightness, resolution, and anatomical structure recognition, whereas the OM was preferred only for natural stereoscopic perception. In equipment functionality (Topic A2), the ExS received higher ratings for mobility, arm operability, stability of video head, and ease of setup, with similar ratings for video head switch usability and foot switch usability. Regarding operative performance (Topics B1-3), the ExS consistently outperformed the OM, including in aspects of surgical field management, ergonomic factors, and cognitive aspects. Team interaction assessments (Topics C1-2) also favored the ExS from both the assistant's and instructor's perspectives. In procedure-specific performance (Topic D), the ExS was rated superior across all steps of CI surgery, with particularly high scores for implant bed creation, cochleostomy, and electrode insertion. Overall, the results demonstrate a clear preference for the ExS over the OM in nearly all evaluated aspects, except for natural stereoscopic perception.

## Discussion

### Summary of the key findings

This study evaluated the feasibility, potential advantages, and limitations of the ORBEYE ExS system compared with the OM in CI surgery in 77 patients (42 ExS and 35 OM). The ExS system demonstrated complete feasibility, with all CI surgeries successfully completed using the ExS alone. While the operative time was significantly longer in the ExS group than in the OM group (162 vs. 130 min, *p* = 0.0051), this difference was associated with a significantly higher rate of resident participation in mastoidectomy procedures (28.6 vs. 8.6%, *p* = 0.0417). When excluding the cases of mastoidectomy by residents, the operating time showed no statistically significant difference between the groups (*p* = 0.1532). Other surgical outcomes and postoperative hearing improvements were comparable between the groups.

The questionnaire results from eight otolaryngologists demonstrated a clear preference for the ExS over the OM across multiple domains, including optical properties, equipment functionality, operative performance, and team interaction. The only aspect in which the OM was preferred was natural stereoscopic perception. The ExS is particularly advantageous for implant bed creation, cochleostomy, and the electrode insertion steps of CI surgery. This study is the first to evaluate the utility of the ORBEYE ExS system in CI surgery, comparing it with conventional OM based on clinical outcomes from a substantial number of cases.

### Utility and limitations of the ExS in otologic surgery

The clinical utility and limitations of various 3D ExS systems for otologic surgery have been reported in several studies (Table 2). The majority of the studies employed the VITOM 3D system (Karl Storz, Tuttlingen, Germany), whereas only three used the ORBEYE system, and four other studies utilized different ExS systems, including the BrightMatter Servo robotic video exoscope system (Synaptive Medical Inc., Toronto, Canada), KINEVO 900 (Carl Zeiss GmbH, Oberkochen, Germany), Aesculap Aeos Robotic Digital Microscope (B. Braun, Kronberg, Germany), and Ainnovi 4 K-3D exoscope otomicrosurgery system (Ainnovi, Zhejiang, China). These 3D ExSs have been evaluated across a range of otologic surgical procedures, including lateral skull base surgery, tympanoplasty, mastoidectomy, stapedotomy, temporal bone dissection, and CI surgery. This broad application suggests the versatility of exoscope technology in these procedures.

**Table 2:**
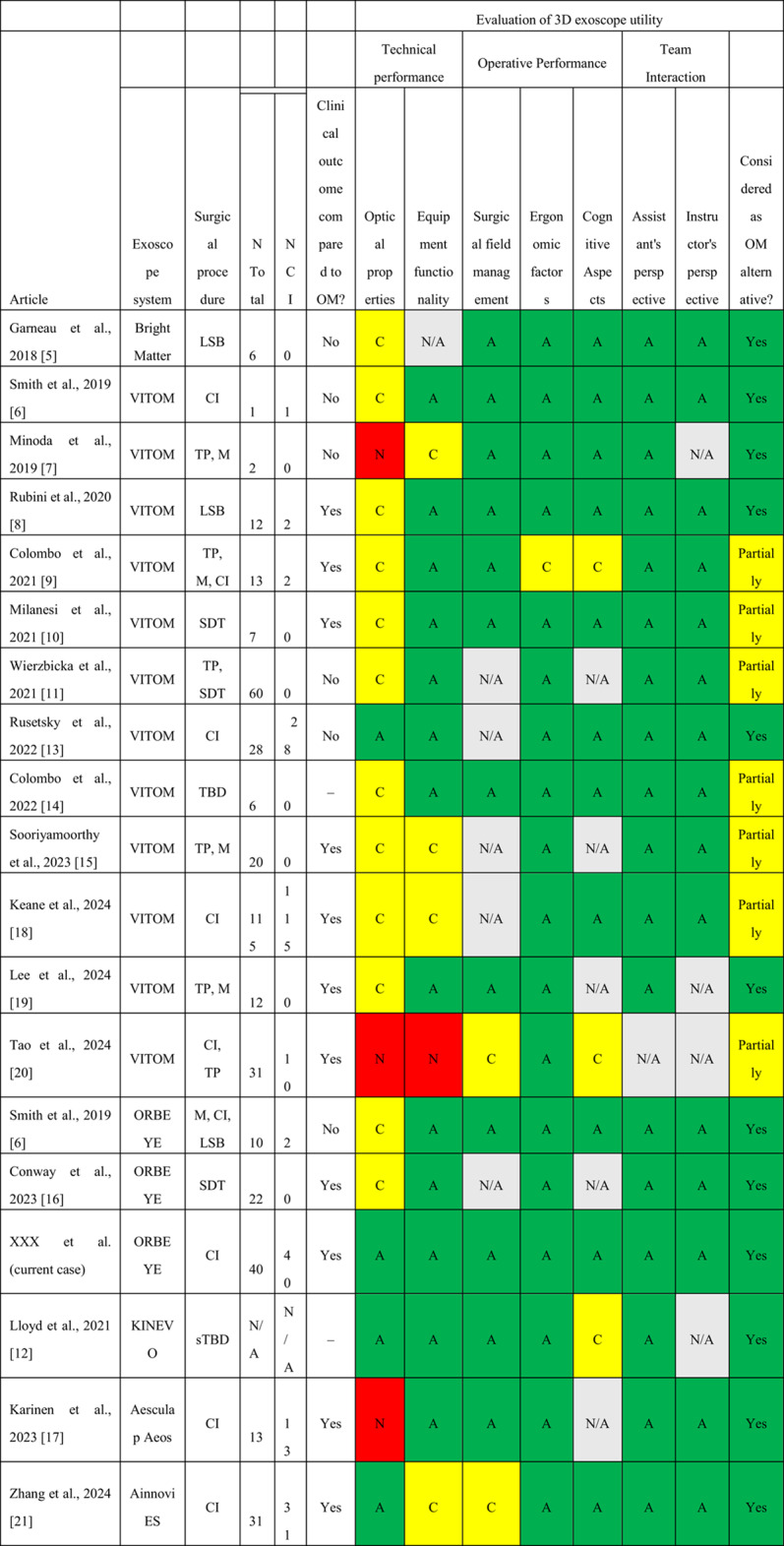
Literature review: Clinical utility and limitations of various 3D exoscopes in otologic surgery

3D, three-dimensional; A, affirmative or acceptable; C, controversial, debatable, or arguable; N, negative, dissenting, or disapproving; N/A, not applicable; CI, cochlear implant surgery; OM, operative microscope; TP, tympanoplasty; M, mastoidectomy; LSB, lateral skull base surgery; SDT, stapedotomy; TBD, temporal bone dissection; sTBD, simulated temporal bone dissection; BrightMatter, BrightMatter Servo robotic video exoscope system (Synaptive Medical Inc., Toronto, Canada); VITOM, VITOM 3D system (Karl Storz, Tuttlingen, Germany); ORBEYE, ORBEYE system (Sony Olympus Medical Solutions Inc., Tokyo, Japan); KINEVO, KINEVO 900 (Carl Zeiss GmbH, Oberkochen, Germany); Aesculap Aeos, Aesculap Aeos Robotic Digital Microscope (B. Braun, Kronberg, Germany); Ainnovi ES, Ainnovi 4K-3D exoscope otomicrosurgery system (Ainnovi, Zhejiang, China)

Among the procedures, CI was the most frequently evaluated procedure across studies, appearing in 7 of the 17 reviewed articles, with several articles focusing exclusively on this procedure. Our current study, utilizing ORBEYE for CI surgery in 42 cases, represents the second-largest cohort after Keane et al.'s report of 115 cases [18]. While Keane et al. conducted their study using VITOM, our research represents the first large-scale evaluation of ORBEYE specifically for CI surgery. This distinction is significant, as it expands the evidence base across different exoscope platforms, potentially identifying system-specific advantages and limitations of this common otologic procedure.

A literature review revealed the advantages and limitations of various 3D ExS systems in otologic surgery. Two otologists (AO and YK) independently reviewed each article to determine whether information regarding each evaluation item related to the 3D ExS utility was present. They classified available information as "A" (affirmative or acceptable), "C" (controversial, debatable, or arguable), or "N" (negative, dissenting, or disapproving). When the reviewers initially disagreed with an item, they conducted a discussion to reach a consensus. Regarding the technical performance, the optical properties showed mixed results. KINEVO, Ainnovi ES, and one of the three ORBEYE studies received an "A" rating, while most VITOM studies received "C" ratings with occasional "A" or "N" ratings. An Aesculap Aeos study and two VITOM studies received "N" ratings. This variability suggests that the optical properties remain a challenge for some 3D exoscope systems. Equipment functionality generally showed positive results across ExS systems, with the majority of studies rating this aspect as "A.” However, among the VITOM studies specifically, three received "C" ratings, and one received an "N" rating, indicating some limitations with this system.

Regarding operative performance, most studies have reported favorable outcomes with the use of the ExS, regardless of the specific system employed. In particular, ergonomic factors were rated as "A" in all but one study. ExS systems allow surgeons to perform heads-up microscope-like surgery [2]. Lin et al. evaluated the surgeon’s posture using the Rapid Upper Limb Assessment (RULA) during middle ear surgeries performed with an ExS, OM, and endoscope and assessed the musculoskeletal disorder risk for each device [22]. Middle ear surgery using the ExS demonstrated the lowest risk profile among the three modalities.

Team interaction was also consistently rated positively, with both assistants’ and instructors’ perspectives receiving acceptable ratings across all the ExS systems evaluated. Although some studies have reported difficulties in stereoscopic perception from the assistant’s position, a 2D sub-monitor positioned directly in front of the assistant helped resolve this issue [13, 23]. In our department, the main monitor is placed approximately 1.5 m in front of the operator and slightly rotated toward the assistant positioned at the patient’s head side, enabling satisfactory stereoscopic visualization from both the operator’s and assistant’s viewpoints (Fig. 1). The educational benefits of the ExS are substantial for both trainees and instructors. Medical students and residents can more easily comprehend otologic surgical anatomy and procedures through ExS [24, 25], while instructors benefit from the comprehensive visualization of the surgical field, allowing for efficient intraoperative guidance with greater confidence [24]. This educational advantage is reflected in our questionnaire results, with all items related to the instructor's perspective receiving high scores. However, it is important to note that, while the ExS lowers barriers to surgical education, this may paradoxically extend operative times due to increased teaching opportunities, as observed in our study and that by Zhang et al. [21].

The potential utility of an ExS as an alternative to a conventional OM varies across studies. While the majority of publications support ExS as a viable alternative to OM, seven studies, all evaluating the VITOM system, indicated only partial suitability, suggesting system-specific limitations. This pattern is particularly noteworthy, as our review of ORBEYE studies consistently reported full suitability as an OM alternative, including in our current study of CI surgery. The varying assessments may also correlate with the surgical procedure; for instance, studies involving stapedotomy and tympanoplasty more frequently resulted in partial suitability ratings than studies focusing solely on CI surgery.

Table 3 summarizes the specific limitations and challenges of the 3D ExS systems in otologic surgery described in the original articles. The VITOM system, which was evaluated in the largest number of studies, demonstrated recurring issues, including image degradation at high magnification (reported in seven studies), insufficient illumination in narrow surgical corridors or deep fields (five studies), and reduced depth perception (three studies). Other VITOM-specific limitations include difficulties in manual focusing, tripod positioning restrictions, and incompatibility with certain surgical equipment. The BrightMatter system was noted for decreased depth perception, increased operative time, and bulky robotic arms. The ORBEYE system was reported to have different limitations, including image inadequacy in deep fields, challenges with deeper middle ear structure visualization, and inferior natural stereoscopic perception, which was also confirmed in our questionnaire results. Other systems, including the KINEVO, Aesculap Aeos, and Ainnovi ES, present unique challenges related to depth perception, image resolution at high magnification, and frequent repositioning requirements, respectively. Taken together, these findings suggest that while ExS technology offers numerous advantages in otologic surgery, system-specific limitations persist, which surgeons should consider when selecting appropriate visualization tools for different otologic procedures.

**Table 3:** Summary of reported specific limitations and challenges with 3D exoscope systems in otologic surgery

Article	Exoscope system	Limitations and challenges
Garneau et al., 2018 [5]	BrightMatter	Decreased depth perception; increased operative time; adjustment required for lack of stereopsis; bulky robotic arm
Smith et al., 2019 [6]	VITOM	Low lighting in narrow surgical corridors; pixelation at high magnification
Minoda et al., 2019 [7]	VITOM	Image degradation at high magnification; lack of autofocus system
Rubini et al., 2020 [8]	VITOM	Lower lighting in narrow corridors; pixelation at high magnification
Colombo et al., 2021 [9]	VITOM	Need for large surgical corridor; image degradation at high magnification
Milanesi et al., 2021 [10]	VITOM	Illumination insufficient in narrow corridors; image degradation at high magnification
Wierzbicka et al., 2021 [11]	VITOM	Insufficient illumination in deep surgical fields; reduced depth perception; image degradation at high magnification
Rusetsky et al., 2022 [13]	VITOM	Tripod positioning initially restricted surgeon hand movement
Colombo et al., 2022 [14]	VITOM	Overexposure of bright structures; high learning curve; manual repositioning limitations
Sooriyamoorthy et al., 2023 [15]	VITOM	Incompatibility with KTP laser glasses; lower levels of magnification; difficult views when bleeding
Keane et al., 2024 [18]	VITOM	Reduced signal-to-noise ratio at higher magnification; manual focus; lack of electromagnetic articulation in the holding arm
Lee et al., 2024 [19]	VITOM	Deterioration of image resolution at high magnification; need for additional refocusing controller; reduced visibility in deep middle ear regions
Tao et al., 2024 [20]	VITOM	Inadequate brightness control; limited depth perception; poor optical contrast and resolution; image degradation at high magnification
Smith et al., 2019 [6]	ORBEYE	Image inadequacy in deep fields in one case; no consistent limitations reported
Conway et al., 2023 [16]	ORBEYE	More difficult assessment of deeper middle ear structures; challenges with transcanal visualization
XXX et al. (current case)	ORBEYE	Inferior natural stereoscopic perception
Lloyd et al., 2021 [12]	KINEVO	Reduced depth perception; limited illumination potential in deeper dissection
Karinen et al., 2023 [17]	Aesculap Aeos	Inferior image resolution at high magnification; pixelation at highest optical zoom
Zhang et al., 2024 [21]	Ainnovi ES	Smaller surgical field at same magnification; more frequent repositioning due to bulky camera head

3D, three-dimensional; BrightMatter, BrightMatter™ Servo robotic video exoscope system (Synaptive Medical Inc., Toronto, Canada); VITOM, VITOM® 3D system (Karl Storz, Tuttlingen, Germany); ORBEYE, ORBEYE™ system (Sony Olympus Medical Solutions Inc., Tokyo, Japan); KINEVO, KINEVO® 900 (Carl Zeiss GmbH, Oberkochen, Germany); Aesculap Aeos, Aesculap Aeos® Robotic Digital Microscope (B. Braun, Kronberg, Germany); Ainnovi ES, Ainnovi 4 K-3D exoscope otomicrosurgery system (Ainnovi, Zhejiang, China)

### Application of the ExS for CI surgery

Only four studies have focused exclusively on the utility of the ExS in CI surgery [13, 17, 18, 21]. The ExS systems employed in these studies included the VITOM (2 studies) [13, 18], Aesculap Aeos (1 study) [17], and Ainnovi ES (1 study) [21]. Keane et al. [18] conducted the largest study to date, examining 115 CI surgeries. They reported that CI surgery was completed solely using the ExS in all but one case, which occurred during the early phase of ExS adoption at their facilities. All ExS systems across these studies provided sufficient image quality to complete CI procedures [13, 17, 21], and no study reported any major complications.

Consistent with these previous reports, in the current study, every CI surgery in the ExS group was completed using only the ORBEYE system. In addition, the results of the questionnaire (Topic D: procedure-specific performance) revealed that the ExS received superior evaluations compared to the OM, even in procedures involving narrow and deep operative fields such as posterior tympanotomy, cochleostomy, and electrode insertion.

### Educational benefits of ExS use in otologic surgery

In otologic surgery using the OM, the stereoscopic view is limited to the operator and assistant, whereas in otologic surgery using the 3D ExS system, the 3D image of the surgical field set by the operator can be shared by all operating room personnel, including trainees, residents, and medical students. For trainees, residents, and medical students, the shared 3D image helps understand the 3D anatomy of complex middle ear structures. In addition, being able to view the surgical field from the operator's perspective helps trainees master the surgical techniques. The ability of instructors to share 3D images enables them to guide trainees with greater confidence and reassurance.

In the present study, the operating time was significantly longer in the ExS group than in the OM group (*p* = 0.0051; Table 1). However, when excluding cases with mastoidectomy by residents, the operating time showed no statistically significant difference between the groups (*p* = 0.1532). This suggests that a longer operating time reflects the educational benefit of ExS use rather than any inferiority of ExS to OM in terms of technical performance or operative efficacy. In fact, residents performed mastoidectomy procedures more frequently in the ExS group than in the OM group (*p* = 0.0417), suggesting that the enhanced visibility provided by the ExS encourages greater trainee participation. Notably, the educational benefits offered by shared 3D images may be one of the most important advantages of the ExS that cannot be achieved with a conventional OM. Further studies are warranted to quantify these educational benefits and to explore the development of effective training methods that utilize the ExS.

### Limitations

Several limitations associated with the present study warrant mention. First, its retrospective design may introduce selection bias, particularly since the ExS was gradually implemented. Second, our evaluation of ExS use in CI surgery was limited to the ORBEYE and may not be generalizable to other ExS systems. Third, the questionnaire responses were based on subjective assessments by otologists, which could introduce recall bias when comparing ExS and OM performance. Fourth, although our sample size of CI surgeries using the ExS was substantial, it may not be sufficiently large to definitively establish the usefulness of the ExS in CI surgery across all scenarios. Finally, the period of ExS utilization may not have been long enough to fully assess the long-term outcomes and learning curve associated with this technology.

## Conclusions

The 4 K-3D ExS ORBEYE demonstrated complete feasibility and comparable surgical outcomes to those of a conventional OM in CI surgery. The longer operative time in the ExS group appeared to reflect educational benefits rather than technical limitations. The questionnaire results from eight otolaryngologists demonstrated a clear preference for ExS over OM across multiple domains, including optical properties, equipment functionality, operative performance, and team interaction. The ExS is particularly advantageous for implant bed creation, cochleostomy, and the electrode insertion steps of CI surgery. Our findings support the use of an ExS as a viable alternative to an OM for CI surgery, with additional benefits, including team interaction and surgical education.

## Data Availability

The datasets generated and analyzed during the current study are available from the corresponding author upon reasonable request.
